# Deciphering the circulating lipidome signature associated with physical performance in gastric cancer patients: an exploratory study

**DOI:** 10.1007/s11306-026-02445-1

**Published:** 2026-05-06

**Authors:** Ana Carolina Pinto, Helena Beatriz Ferreira, Samuel Barbosa, Tiago Sousa, Tânia Melo, Lúcio Lara Santos, Rita Ferreira, Daniel Moreira-Gonçalves, Maria do Rosário Domingues

**Affiliations:** 1 LAQV-REQUIMTE, Department of Chemistry, University of Aveiro, Campus Universitário de Santiago, 3810-193 Aveiro, Portugal; 2 CESAM, Centre for Environmental and Marine Studies, Department of Chemistry, University of Aveiro, Campus Universitário de Santiago, 3810-193 Aveiro, Portugal; 3 Experimental Pathology and Therapeutics Group, Research Center (CI-IPOP)/RISE@CI-IPOP (Health Research Network), Portuguese Oncology Institute of Porto (IPO-Porto)/Porto Comprehensive Cancer Center (P.CCC), 4200-072 Porto, Portugal; 4 CIAFEL, Research Center in Physical Activity, Health and Leisure, Faculty of Sport, University of Porto, 4200-450 Porto, Portugal

**Keywords:** Lipidomics, Mass spectrometry, Cancer, Physical activity

## Abstract

**Introduction:**

Physical performance strongly influences peri-treatment outcomes in gastric cancer (GC), yet simple molecular markers reflecting functional status are lacking. Lipidomics may help identify circulating biomarkers linked to physical fitness.

**Objectives:**

To assess whether physical performance is associated with distinct plasma lipidomic profiles in GC patients.

**Methods:**

Nineteen male GC patients (60–75 years) from the PROTECT trial were classified as high- (HighP) or low-performance (LowP) based on the 6-min walk test. Plasma lipidomics (LC–MS/MS) quantified 232 lipid species and a total of 25 fatty acids were quantified by gas chromatography-MS. Multivariate and univariate analyses, group comparisons, and correlations examined associations with clinical, anthropometric, and fitness parameters.

**Results:**

Lipid profiles differed by performance status. HighP patients showed higher phosphatidylinositol (PI 36:2) and trends toward increased plasmalogen phosphatidylethanolamines (PE), whereas sphingomyelin (SM 43:2) was higher in LowP patients. Plasmenyl-PE species correlated positively with functional tests, muscle mass, body mass index, and nutritional status; SM 43:2 correlated negatively. Acylcarnitines showed minimal associations.

**Conclusion:**

GC patients with different physical performance status display distinct circulating lipid signatures. PI 36:2, PE plasmalogens, and SM 43:2 species appear linked to physical fitness, suggesting potential value as preoperative biomarkers. Validation in larger cohorts is warranted.

**Supplementary Information:**

The online version contains supplementary material available at 10.1007/s11306-026-02445-1.

## Introduction

Despite significant declines in mortality over the past century, gastric cancer (GC) remains the fourth leading cause of cancer-related deaths worldwide and is often overlooked (Yang et al., 2023). Preoperative chemotherapy with FLOT (fluorouracil, leucovorin, oxaliplatin, and docetaxel) has become the standard of care for patients with locally advanced GC. However, despite the improved efficacy of newest chemotherapy regimens leading to improved oncological outcomes, treatment-related toxicities remain a significant concern. In fact, adverse events of preoperative chemotherapy may lead to the significant compromise of physiological reserve, decreasing the ability of the patients to tolerate surgical stress and increasing the risk for postoperative complications (Christodoulidis et al., 2023; Wu et al., 2021). As a result, thorough assessment of patients’ tolerance to anticancer treatment is crucial for risk stratification and informed decision-making in GC patient management. Physical fitness is a strong surrogate of physiological reserve, with low levels of aerobic capacity or strength associated with poor tolerance and greater toxicity for chemotherapy. Moreover, low physical fitness has been shown to be a string. Therefore, patients presenting with low fitness levels at the time of diagnosis experience lower survival at one-year postoperatively (West et al., 2021). Despite the clear prognostic value of fitness assessment, the implementation of muscle strength and functional tests in routine clinical practice is often limited by system-level barriers, staff training, and prioritisation. Therefore, identifying simple and practical molecular tests representative of the patients’ physical status could provide a valuable tool for clinical decision-making.

The analysis of biomolecular profiles can provide valuable insights into an individual's overall health. Lipids are crucial molecules for a wide range of biological processes, including energy generation and storage, cell signalling, and hormone production (Han & Gross, 2022; Yetukuri et al., 2007). In cancer, lipids are important in all the processes essential for tumour development, including cell growth and metabolism (essential for rapidly proliferating cancer cells, also as major components of cell membranes) (Mika et al., 2020; Ren et al., 2006; Szlasa et al., 2020). Mass spectrometry (MS)-based lipidomics enables large-scale, comprehensive profiling of the cellular or circulating lipidome (Lagarde et al., 2003; Spener et al., 2003; Yetukuri et al., 2007), allowing the identification of over a hundred lipid species in plasma or serum samples (Gaggini et al., 2021; Høeg et al., 2020; Lehmann et al., 2010; Osuna‐Prieto et al. 2023; Pataky et al., 2024; A. Wang et al., 2023a, 2023b; Haifeng Zhang et al., 2023), including triacylglycerols (TGs), phospholipids (PLs), sphingolipids, such as sphingomyelin (SM), cholesterol esters, free fatty acids, and bile acids, among other minors. Physical activity has been associated with distinct lipidome profiles. For instance, physically active individuals exhibit significantly higher baseline concentrations of circulating 12,13-dihydroxyoctadecenoic acid compared to those with a sedentary lifestyle, with levels further increasing immediately after an acute bout of exercise (Stanford et al., 2018). Additionally, lyso-phosphatidylcholine (LPC,15:0) has been positively associated with high levels of physical activity (Hoshi et al., 2022). LPC species have been reported to enhance the release of arachidonic acid, inflammatory cytokines, and growth factor (Aiyar et al., 2007). However, lower LPC levels have been linked to increased arterial stiffness and impaired endothelial function (Paapstel et al., 2018).

Despite the growing interest in lipidomics for identifying physical activity-related molecular profiles, its application remains limited (Ong et al., 2023), particularly in cancer settings. Therefore, this study aimed to investigate the impact of physical performance on the plasma lipidome of adult male patients newly diagnosed with GC, using a LC–MS-based approach to identify potential lipid molecular markers of physical performance. To our knowledge, this is the first study to characterize the circulating lipidome profile of GC patients in relation to physical performance, offering valuable insights into the health benefits of physical activity and paving the way for the identification of novel markers of physical status.

## Methods

### Study population

Plasma samples were collected from GC patients enrolled in the PROTECT clinical trial, which aims to enhance treatment outcomes by incorporating prehabilitation programs, including structured exercise regimens, into a multidisciplinary approach for managing GC. This project was approved by IPO-Porto Ethics Committee (CES 145/020) and adheres to the ethical standards outlined in the 1964 Declaration of Helsinki and its subsequent amendments. PROTECT inclusion criteria are: patients first-diagnosed with advanced GC (stages III–IV), age > 18 years, and mentally/physically able to consent and participate. Subjects meeting the inclusion criteria were recruited to the PROTECT study prior to cancer treatment. Baseline examination includes anthropometric and fitness measurements, nutritional assessment, self-administered questionnaires on physical activity, and blood collection for standard biochemical analysis and lipidomics. Plasma samples were stored at − 80 °C until further use.

Nineteen male patients, aged 60 to 75, diagnosed with GC and not yet undergoing treatment, were randomly selected for inclusion in this study. Demographic and clinical information [including age, body weight, arm perimeter, tumour stage, medical history, nutritional assessment (PG-SGA), biochemical data, and fitness metrics (including 6-min walking test (6MWT), sit-to-stand test, IPAQ level of activity)] is included in the Supplementary Table S1. All data were anonymised for analysis.

### Lipid extraction and quantification

Lipids were isolated from plasma samples by the Bligh & Dyer method, in random order, independent of group. Briefly, 50 μL of plasma were divided into two glass tubes, and 1 mL of Milli-Q water, 2.5 mL methanol and 1.25 mL dichloromethane (DCM) were added. The samples were vortexed for 2 min, left on ice on the Orbital Shaker for 30 min, and after vortexed for 30 s. Next, 1.25 mL DCM and 1.25 mL Milli-Q were sequentially added, each followed by 1 min vortexing, resulting in phase separation in each tube. All glass tubes were centrifuged (2000 rpm, 10 min), and the lower organic phase was collected into a new tube. Residual material was re-extracted with 1.88 mL DCM, vortexed (2 min), centrifuged (10 min, 2000 rpm), and the organic phase was combined with the first lipid extract. The extracts were dried under a stream of nitrogen, and the lipid content was resuspended with 300 µL DCM and transferred to dark vials. This process (resuspension and transfer) was repeated twice, after which the extracts were dried and stored at -20ºC until further analysis.

Total PL content was determined by phosphorus assay as previously reported (H. B. Ferreira et al., 2023).

### LC–MS/MS

The lipid extracts obtained from plasma samples were resuspended in DCM to have a PL concentration of 1 μg PL/μL. Subsequently, in a vial with a micro insert, 10 μL of each sample, 8 μL of a mixture of internal standards and 82 μL of a solvent mixture consisting of IPA/MeOH (1:1, v/v) were added. The internal standard mixture contained 0.04 μg of PC 14:0/14:0, 0.04 μg of PE 14:0/14:0, 0.024 μg of phosphatidylglycerol (PG) 14:0/14:0, 0.08 μg of PI 16:0/16:0, 0.08 μg of PS 14:0/14:0, 0.16 μg of phosphatidic acid (PA, 14:0/14:0), 0.04 μg of LPC 19:0, 0.04 μg of SM d18:1/17:0, 0.08 μg of Cer d18:1/17:0, and 0.16 μg of cardiolipin (CL, 14:0/14:0/14:0/14:0). The internal standard mixture was used as suitability test sample, it was dissolved in the solvent mixture (IPA/MeOH, 1:1 v/v) and did not contain sample matrix.

Lipids were separated by reversed-phase liquid chromatography using an Ascentis Express90 Å C18 HPLC column (15 cm × 2.1 mm; 2.7 μm, Supelco) in an HPLC system (Ultimate 3000 Dionex, Thermo Fisher Scientific, Bremen, Germany) with an autosampler and coupled online to a Q-Exactive hybrid quadrupole-Orbitrap mass spectrometer (Thermo Fisher Scientific, Bremen, Germany). LC–MS/MS conditions and settings were the same as reported elsewhere (Guerra et al., 2024). Solvent blank samples were also acquired. LC–MS/MS data were processed using Lipostar software (Molecular Discovery Ltd., version 2.1.5 × 64) (Goracci et al., 2017). This software was used for raw data import, peak detection, identification and integration. Lipid assignment and identification were made against a database created from the LIPID MAPS structure database (version April 2024), which was then fragmented using the DM Manager Module in Lipostar, according to Lipostar fragmentation rules. The raw files were imported directly and aligned using the following settings: briefly, automatic peak picking was performed with the SDA smoothing level set to high and a minimum S/N ratio of 3 with an automatic signal filtering threshold. The isotope clustering settings were configured to 10 ppm with a retention time (RT) tolerance of 0.2 min in an automated fashion. The MS/MS filter was applied to keep only features with MS/MS spectra for identification. Lipid identification was made according to the following parameters: 5 ppm precursor ion mass tolerance and 10 ppm product ion mass tolerance. The automatic approval was performed to keep structures with a quality of 3 − 4 stars. The areas under the curve (AUC) were measured through chromatographic peak integration. Peak integration and lipid identification were manually reviewed for a subset of lipid species, with confirmation through evaluation of exact mass accuracy, MS/MS fragmentation patterns, characteristic ions, and other class-specific spectral features.

Internal standards were used to perform quantification of the lipid molecular species by pairing each standard to its respective internal standard, as previously reported (Ferreira et al., 2021, 2023). Relative quantification was performed by exporting the peak area values to an Excel spreadsheet. For data normalisation, the peak areas of the extracted ion chromatograms (XIC) for each lipid class were divided by the peak areas of the assigned internal standards selected (Supplementary Table S2).

### GC–MS/MS analysis

Fatty acids (FA) analysis was performed by GC–MS after the transmethylation of PL-enriched extracts, as routinely used in our laboratory (Guerra et al., 2021; Sousa et al., 2016), in order to convert the FAs present in these extracts into their corresponding fatty acid methyl esters (FAMEs). This transmethylation (or derivatization) allowed for an easier FA analysis, since FAMEs are generally more stable and volatile than FAs. A hexane solution containing FAMEs and methyl nonadecanoate (FA 19:0), as internal standard, was injected into a GC–MS system equipped with a DB-FFAP column. The operation conditions included helium as the carrier gas, an inlet temperature of 220ºC, detector temperature set at 230ºC, and an injection volume of 2 μL. The oven temperature was programmed to vary from 58ºC to 225ºC, and data was acquired using the GCMS5977B/Enhanced MassHunter software. The acquired data were analysed using the software Agilent MassHunter Qualitative Analysis 10.0. FA identification was achieved by the comparison of the MS spectra with the chemical database NIST library and “The Lipid Web” (Lipidomics Gateway, n.d.). Retention times (RT) and MS spectra of FAME standards (Supelco 37 Component FAME Mix, Sigma-Aldrich, Darmstadt, Germany) were also considered. FA quantification was conducted using calibration curves obtained from FAME standards under the same instrumental conditions.

### Statistical analysis

Data on patients’ characteristics and esterified FA analysis are expressed as mean ± standard deviation (SD) for each experimental group. Normality was assessed using the Shapiro–Wilk test. Group comparisons were performed using independent-samples t-test for normally distributed data, and the Mann–Whitney U test for non-normally distributed data. Multivariate statistical analyses of the lipidomic dataset (LC–MS) were performed in MetaboAnalyst v.6.0 (J. Xia et al., 2009). Missing values for lipid molecular species were imputed as 1/5 of the minimum positive value detected in the dataset. Regarding data normalisation in Metaboanalyst, the data underwent a log_10_ transformation and was auto-scaled. Unsupervised principal component analysis (PCA) and supervised partial least squares discriminant analysis (PLS-DA) were also performed in MetaboAnalysist, with patient grouping based on the 6MWT. Hierarchical clustering analysis was additionally performed and visualized through a heatmap of the lipid abundance matrix, illustrating inter-individual variability and overall lipid patterns. Given the small, imbalance and biologically heterogeneous nature of the cohort, univariate non-parametric analysis was adopted in MetaboAnalyist, with results illustrated through a volcano plot. Lipid species with the highest discriminatory power were selected for further analysis, and statistical results were further confirmed by independent analysis in GraphPad Prism (v.9.0.2), providing an additional layer of validation. To account for the potential confounding effect of pharmacological treatment, ANCOVA regression models were constructed for each lipid species individually, integrating statin and metformin use as covariates, with model assumptions verified through Shapiro–Wilk normality testing of unstandardized residuals (homoscedasticity) and Levene's test for homogeneity of variances. Associations between clinical, anthropometric and fitness parameters and circulating lipid species were evaluated using Pearson correlation coefficient in SPSS Statistics (IBM SPSS Statistics, version 29.0.0.0). Statistical significance was defined as *p*-value ≤ 0.05.

## Results

To assess the impact of GC patients’ performance capacity on the circulating lipidome, patients were categorised into two groups based on their 6MWT values, a marker of functional capacity and aerobic endurance, relative to the 50th percentile (P50) established for the Portuguese population (Marques et al., 2014). Considering the total of 19 patients, 6 patients were assigned to the High Performance (HighP; > P50) group and the other 13 patients were assigned to the Low Performance (LowP; < P50) group. Comparison of baseline characteristics of these groups are summarised in Supplementary Table S3. The tumour development stage was similar in both groups. No significant differences were observed between groups with respect to patient age, IPAQ and PG-SGA scores or average daily steps. In contrast, the HighP group showed significantly greater handgrip strength and sit-to-raise performance (p < 0.01), consistent with the trend observed for the 6MWT. No significant differences between groups were observed for the standard biochemical parameters (*p*-value > 0.05), except for total cholesterol and LDL levels, which were unexpectedly higher in HighP group. However, only one patient in the HighP group, compared with seven into LowP group, was receiving statin treatment, and four patients in the LowP group had diabetes managed with metformin, with none requiring insulin therapy.

### Lipidome differences between high- and low-performance patients

LC–MS and MS/MS analysis allowed the identification of 232 different lipid species belonging to 13 different (sub)classes, namely phosphatidylcholines (PC) including diacyl, alkyl–acyl and alkenyl–acyl species, lyso PC (LPC), phosphatidylethanolamine (PE) including diacyl, alkyl–acyl and alkenyl–acyl species, lyso PE (LPE), phosphatidylinositol (PI), phosphatidylserine (PS), SM, ceramide (Cer), acylcarnitine (CAR), cholesteryl ester (CE), diacylglycerol (DG), TG and the glycosphingolipids (galactosylceramide (GalCer) and lactosylceramide (LacCer)) (Supplementary Table S4).

**Fig. 1:**
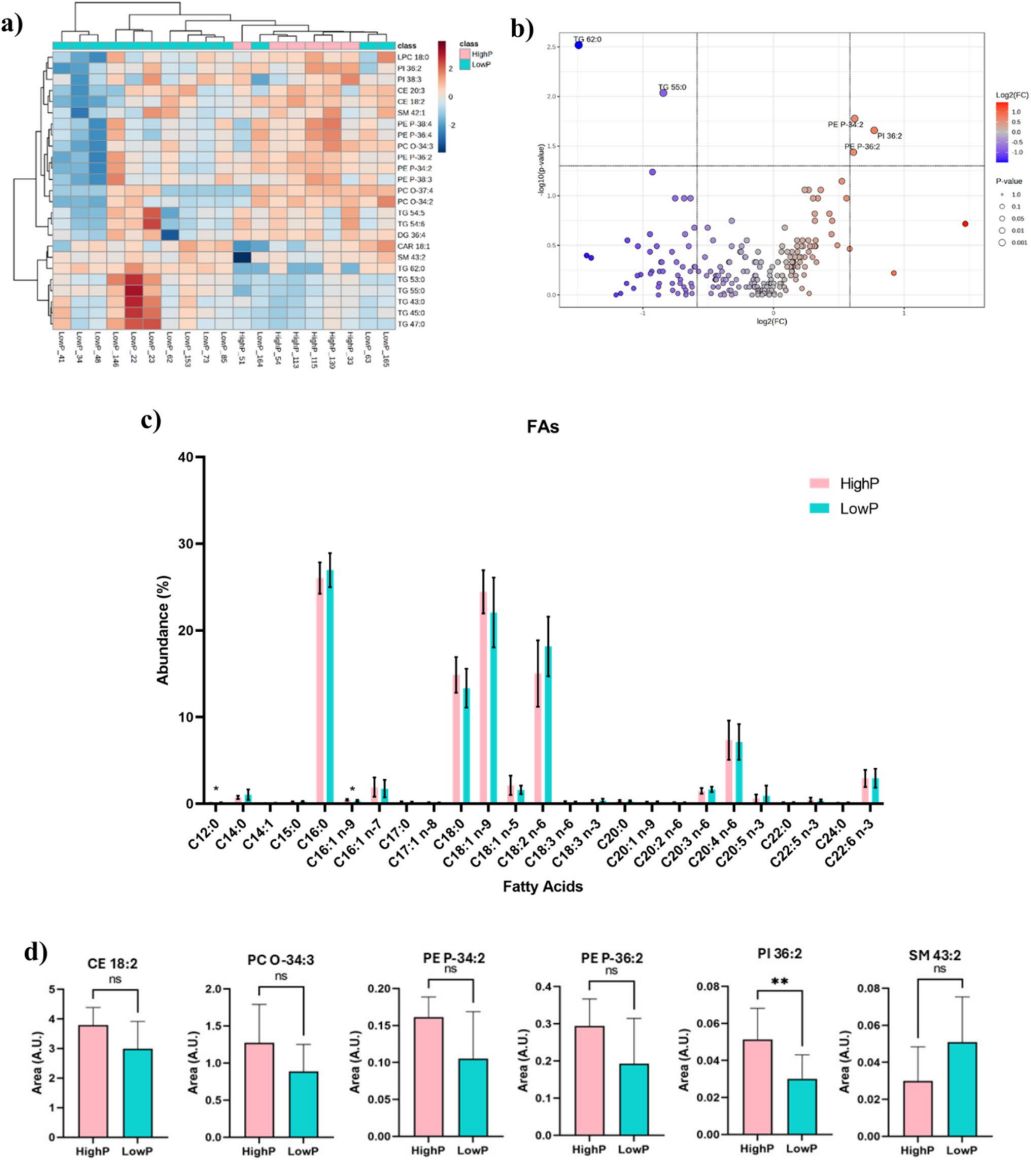
**a** Two-dimensional hierarchical clustering heatmap of the 25 most discriminating lipid molecular species between HighP and LowP groups. Relative abundance levels are shown on the color scale, with numbers indicating the fold difference from the overall mean. The clustering of the LowP (blue) and HighP (pink) groups is represented by the dendrogram at the top. The clustering of individual lipid molecular species is represented by the dendrogram on the left. **b** Volcano plot of circulating lipid species between HighP and LowP groups. Each dot represents an individual lipid species, with dot colour reflecting the direction of the fold change (red, higher abundance in HighP; blue, higher abundance in LowP). The horizontal and vertical dashed lines represent the significance threshold (p ≤ 0.05) and fold change thresholds (log2(FC) = ± 1), respectively. Labelled species exceed both thresholds simultaneously. **c** Plasma fatty acid profile of gastric cancer patients analyzed by GC–MS. Values are means ± standard deviation. The * symbol represents statistically significant differences (*p*-value ≤ 0.05) between groups. **d** Independent statistical analysis of the 6 most discriminating lipid molecular species between HighP and LowP groups. The ** symbol represents statistically significant differences (*p*-value ≤ 0.01) between groups

The effect of physical performance, as assessed by the 6MWT, on the circulating lipidome was analysed. The differences in the lipid profiles of the patient groups were assessed using multivariate statistical analysis. PCA and PLS-DA plots of the 19 samples revealed no clear separation between HighP and LowP groups, consistent with the biological heterogeneity expected in a small clinical cohort (Fig. S1). Additionally, hierarchical clustering analysis (HCA) of the lipid data sets revealed distinct patterns of lipid abundance across patients, illustrating inter-individual variability within groups (Fig. 1a). The heatmap displays the top 25 lipid species ranked by ANOVA p-values generated in MetaboAnalyst as an exploratory visualisation and feature selection step; q-values were subsequently applied for multiple testing correction and the 25 features shown in Fig. 1a represent those with the lowest q-values. The two-dimensional dendrogram reflects the most discriminating lipid species contributing to group differentiation based on 6MWT performance levels. The upper dendrogram revealed an incomplete separation between groups, with some LowP samples interspersed among HighP patients, consistent with the biological heterogeneity of the cohort. The second dimension shows two principal clusters: the first group includes 17 lipid species, namely 5 PE, 3 PC, 2 PI, 2 CE, 2 TG, 1 LPC, 1 SM, and 1 DG, which tended towards higher relative abundance in the HighP patients; on the other hand, the second group has 8 different lipid species, which include 6 TG, 1 CAR, and 1 SM, which showed a tendency to be in lower levels in the HighP group.

Univariate non-parametric analysis was performed in MetaboAnalyst and visualized through a volcano plot illustrating the distribution of lipid species according to their fold change and statistical significance between groups (Fig. 1b). Despite the small and biologically heterogeneous nature of the cohort, several lipid species showed noteworthy differences between groups. TG 62:0 and TG 55:0 emerged as the most statistically significant species, both displaying lower abundance in HighP patients (negative Log2(FC), depicted in blue). Conversely, PE P-34:2, PE P-36:2 and PI 36:2 were located on the right side of the plot with positive changes, indicating higher abundance in HighP patients and suggesting a potential association between these phospholipids and physical performance status.

The identification and quantification of the esterified FA profile of the total lipid extract were performed by GC–MS analysis of fatty acid methyl esters. A total of 24 FA were quantified. The major FA identified were C16:0, C18:0, C18:1*n*-9, C18:2*n*-6, C20:4*n*-6, and C22:6*n*-3 (Fig. 1c). Comparative group analysis showed higher levels of C12:0 in LowP and C16:1*n*-9 in HighP patients.

To further validate these observations, independent *t-*test/Mann–Whitney analyses were conducted on the 17 non-TG lipid species retrieved from HCA. Of these, PI 36:2 showed significant differences (*p*-value = 0.0074), confirming its higher abundance in HighP subjects and corroborating the volcano plot findings. Additionally, several species demonstrated tendencies toward higher abundance in HighP patients, including CE 18:2 (*p*-value = 0.0711), PC O-34:3 (*p*-value = 0.0758), PE P-34:2 (*p*-value = 0.0553), PE P-36:2 (*p*-value = 0.0781), reinforcing the potential association between ether-linked phospholipids and better physical performance. Notably, SM 43:2 (*p*-value = 0.0911) showed an opposite trend with higher abundance in LowP patients (Fig. 1d). The non-significant differences are shown in Fig. S2. Following adjustment for statin and metformin use in ANCOVA regression models, the significance of PI 36:2 was confirmed in HighP patients (p = 0.008), with three additional lipid species reaching statistical significance: PI 38:3 (p = 0.015), PE P-34:2 (p = 0.042) and PE P-36:2 (p = 0.041), all showing higher abundance in HighP patients. Conversely, SM 43:2 was confirmed as significantly elevated in LowP patients (p = 0.042; Supplemental Table S5).

To provide mechanistic clues regarding the potential biological relevance of the identified lipid species and guide future hypothesis-driven experimental validation, Pearson correlation analysis between lipidomic and clinical or anthropometric data was performed (Table 1). The sit-to-stand test showed the highest number of significant correlations among the functional performance measures, likely reflecting its closer functional overlap with 6MWT. Positive associations were observed for some of the identified plasmalogens and CAR 18:1, while SM 43:2 showed a negative correlation with this functional performance test. Several plasmalogens (PE P-34:2; PE P-36:2; PE P-36:4; PE P-38:3; PE P-38:4) also showed a positive correlation, or sometimes a tendency for a positive correlation, with the Nutritional Risk Index (NRI), a screening tool for assessing nutritional status, as well as to the handgrip test and circumferences. As for the identified plasmanyl-phosphatidylcholines (alkyl acyl PC, PC O-), a positive correlation was observed with both arm and calf circumferences, suggesting a relevance of these lipids in physical parameters; on the other hand, these PCs were also positively correlated with BMI and weight of the GC patients, with PC O-34:2 being negatively correlated with a non-intentional weight loss. Lastly, the identified CEs were positively correlated with LDL-c and total CHOL, reflecting their shared role in cholesterol transport and lipid metabolism.

**Table 1:** Pearson correlation coefficients (r) between plasma lipid species and clinical and anthropometric parameters

			95% Confidence Intervals (2-tailed)^a^	
	Pearson Correlation Coefficient	Sig. (2-tailed)	Lower	Upper
Age vs. PE P-34:2	-.407	.084	-.727	.058
Age vs. PE P-36:2	-.405	.086	-.726	.060
Age vs. PE P-38:3	-.456	.**050**	-.754	-.003
Age vs. FA 16:1*n*-9	-.429	.067	-.739	.003
Arm circumference vs. PC O-34:2	.595	.**007**	.193	.826
Arm circumference vs. PC O-37:4	.605	.**006**	.209	.831
Arm circumference vs. FA 12:0	.553	.**014**	.131	.805
Calf circumference vs. PC O-34:2	.603	.**006**	.205	.830
Calf circumference vs. PC O-37:4	.468	.**043**	.017	.761
Calf circumference vs. FA 16:1*n*-9	-.390	.099	-.717	.079
CRP vs. SM 43:2	.499	.**035**	.042	.783
Handgrip vs. LPC 18:0	.413	.079	-.050	.730
Handgrip vs. PC O-34:2	.401	.089	-.065	.724
Handgrip vs. PE P-34:2	.540	.**017**	.114	.799
Handgrip vs. PE P-36:2	.550	.**015**	.127	.803
Handgrip vs. PE P-36:4	.447	.055	-.009	.749
Handgrip vs. PE P-38:3	.457	.**049**	.003	.755
Handgrip vs. PE P-38:4	.402	.088	-.064	.724
HDL-c vs. CE 18:2	.409	.092	-.072	.736
HDL-c vs. SM 43:2	-.526	.**025**	-.797	-.078
BMI vs. PC O-34:2	.612	.**007**	.203	.839
BMI vs. PC O-37:4	.757	.**0003**	.448	.904
BMI vs. PE P-36:4	.450	.061	-.021	.758
LDL-c vs. CE 18:2	.723	.**001**	.386	.890
LDL-c vs. CE 20:3	.733	.**001**	.405	.894
LDL-c vs. SM 42:1	.436	.071	-.039	.750
LDL-c vs. FA 16:1*n*-9	.453	.059	-.017	.759
sit to stand test (30 s) vs. CAR 18:1	-.469	.**043**	-.761	-.018
sit to stand test (30 s) vs. PC O-34:3	.431	.066	-.029	.740
sit to stand test (30 s) vs. PE P-34:2	.553	.**014**	.133	.805
sit to stand test (30 s) vs. PE P-36:2	.605	.**006**	.209	.831
sit to stand test (30 s) vs. PE P-36:4	.479	.**038**	.032	.766
sit to stand test (30 s) vs. PE P-38:3	.497	.**031**	.053	.775
sit to stand test (30 s) vs. PE P-38:4	.551	.**015**	.129	.804
sit to stand test (30 s) vs. PI 36:2	.565	.**012**	.148	.811
sit to stand test (30 s) vs. SM 43:2	-.627	.**004**	-.842	-.241
Non-intentional weight loss vs. DG 36:4	-.416	.076	-.732	.047
Non-intentional weight loss vs. PC O-34:2	-.520	.**022**	-.788	-.086
NRI vs. LPC 18:0	.480	.**038**	.033	.767
NRI vs. PE P-34:2	.601	.**007**	.202	.829
NRI vs. PE P-36:2	.610	.**006**	.216	.834
NRI vs. PE P-36:4	.446	.056	-.010	.749
NRI vs. PE P-38:3	.468	.**043**	.018	.761
NRI vs. PE P-38:4	.432	.065	-.028	.741
Total CHOL vs. CE 18:2	.703	.**001**	.352	.881
Total CHOL vs. CE 20:3	.643	.**004**	.252	.854
Weight vs. CE 18:2	.474	.**047**	.009	.770
Weight vs. PC O-34:2	.704	.**001**	.354	.881
Weight vs. PC O-37:4	.580	.**012**	.155	.824
Weight vs. PE P-34:2	.480	.**044**	.016	.773
Weight vs. PE P-36:2	.439	.068	-.035	.752
Weight vs. FA 16:1*n*-9	-.409	.092	-.735	.072

^a^Estimation is based on Fisher's r—to—z transformation with bias adjustment

Significant correlations are highlighted in bold (p < 0.05)

Legend: CAR, fatty acylcarnitine; CE, cholesteryl ester; CHOL, cholesterol; DG, diacylglycerol; FA, fatty acid; LPC, lysophosphatidylcholine; PC, phosphatidylcholine; PE, phosphatidylethanolamine; SM, sphingomyelin

## Discussion

The present exploratory study highlights the impact of physical performance on the circulating lipidome signature in GC patients. While the physiological benefits of regular physical activity are well established, the underlying mechanisms remain incompletely understood in cancer settings. Moreover, a robust molecular signature of physical performance that could aid in predicting GC treatment outcomes has yet to be identified. The present study, although exploratory in nature, provides a first characterization of specific lipid species differentially distributed according to fitness status in GC patients, offering a preliminary molecular framework that may contribute to a better understanding of the systemic adaptations associated with physical performance in this population.

Among the lipid species identified, PI 36:2 emerged as one of the most statistically robust discriminators between HighP and LowP GC patients, retaining significance after adjustment for statin and metformin use in ANCOVA models. Phosphatidylinositols are key membrane components and precursors of phosphoinositides, a family of bioactive signalling lipids that regulate critical cellular processes including metabolic homeostasis through the PI3K/Akt signalling (Balla, 2013). PI 36:2 is the second most abundant PI species in human plasma (T. Xia et al., 2020), being present exclusively in lipoproteins and not in plasma lipoprotein-free fraction (Dashti et al., 2011), suggesting that its circulating levels reflect lipoprotein phospholipid remodelling capacity. PI 36:2 has previously been identified as a component of a lipid ratio (together with PC (18:0/20:4) capable of explaining the cardiovascular risk reduction associated with pravastatin and simvastatin treatment, independent of LDL-cholesterol changes (Schooneveldt et al., 2021). The fact that PI 36:2 remained significantly elevated in HighP patients even after adjusting for statin use suggests that its association with physical performance is independent of this pharmacological effect, pointing instead to a physiologically driven association with metabolic fitness that may reflect a better capacity for lipoprotein-mediated phospholipids delivery to metabolically active tissues. To the best of our knowledge, no previous study has reported an association between PI 36:2 and physical performance or cancer, rendering this hypothesis-generating finding to be validated in larger, prospectively designed cohorts.

PE P-34:2 and PE P-36:2 were also found significantly elevated in HighP patients, and when focusing on the lipids showing the strongest correlations with fitness parameters, limbs circumference and body weight, these plasmenyl-PEs emerged as the most relevant species for functional performance. Plasmalogens, including the plasmenyl-PE (PE P-), are structurally similar to phospholipids but differ at the sn—1 position by containing a vinyl-ether rather than an ester bond. This unique chemical structure makes plasmalogens highly reactive to reactive oxygen species, enabling them to serve as endogenous antioxidants that protect membrane lipids and other biomolecules from oxidative damage (Dorninger et al., 2020; Honsho & Fujiki, 2019; Zoeller et al., 1988). Reduced plasmalogens levels may suggested impaired endogenous antioxidant system, and in particular decreased PE P- have been reported in several conditions, including various cancers (Curran et al., 2025). In our exploratory study, the higher abundance of PE P- species in HighP subjects, particularly PE P-34:2 and PE P-36:2, may be associated with a better preserved antioxidant capacity, as previously reported in response to exercise training-induced physiological stress (Bailey et al., 2007; Y. Wang et al., 2023a, 2023b). Although no significant differences in IPAQ scores were observed in this cohort of GC patients, a trend toward higher average daily step counts was noted, alongside higher fitness levels (Supplemental Table S3).

No specific focus was given to TG species identified in the volcano plot (Fig. 1b), as their biological roles remain poorly understood; however, TG 62:0 and TG 55:0 emerged as significant discriminators, both showing lower abundance in HighP patients. Although these very long-chain saturated TGs are poorly characterized, previous evidence suggest that TGs containing saturated fatty acyl chains do not accumulate following exercise, likely due to their lipotoxic effects and limited capacity for efficient storage (Y. Zhang et al., 2025). The higher circulating abundance of TG 62:0 and TG 55:0 in LowP patients may reflect impaired lipid mobilisation and oxidation, potentially compounded by tumour-driven impairment of lipid oxidative capacity in the cancer setting.

SM 43:2 was also significantly higher in LowP patients after adjustment for statin and metformin use in ANCOVA models (Supplementary Table S5) and showed a positive correlation with CRP levels (Table 1), suggesting that its higher abundance in this group of patients may be embedded within a broader pro-inflammatory context. SM metabolism is linked to the generation of ceramide through sphingomyelinase-mediated hydrolysis, a pathway known to be activated by pro-inflammatory cytokines including TNF-α and IL-1β (Chen et al., 2026). Sphingomyelinase hydrolyses membrane SM into ceramide, which increases oxidant activity in muscle cells, depresses maximal muscle force and accelerates fatigue, with serum sphingomyelinase activity being elevated in conditions of systemic inflammation where muscle oxidants are increased and contractile function is diminished (L. F. Ferreira et al., 2010). While the exploratory nature of the present study precludes any causal inference, these findings provide a compelling hypothesis warranting further mechanistic investigation in larger cohorts.

Interestingly, our exploratory study did not identify any significant associations between CAR and fitness parameters, aside from a negative correlation between CAR 18:1 and the sit-to-stand test (Table 1). This data is consistent with earlier reports indicating that, among elderly men, higher acylcarnitine factor scores are associated with poor objectively measured physical performance (including reduced gait speed and chair stand ability) (Lum et al., 2011). Higher levels of long-chain CAR are often indicative of impaired mitochondrial fatty acid oxidation and reduced efficiency of energy metabolism, both of which contribute to decreased physical performance (Lum et al., 2011; McCoin et al., 2015).

When considering how circulating lipids travel from source to target cells, and thereby mediate the effects of physical activity (Gakis et al., 2023), two main transport systems are typically discussed: lipoproteins and extracellular vesicles (EVs). Notably, PI, PE and SM species have been shown to be present exclusively in lipoprotein fractions (Dashti et al., 2011), suggesting that the observed differences in PI 36:2 and SM 43:2 between HighP and LowP patients may reflect alterations in lipoprotein lipid remodelling. In contrast, our results showed that only CEs were positively correlated with LDL, likely reflecting the high CE content characteristic of these lipoproteins. EVs represent an additional transport system, acting as membrane-bound carriers of biological information capable of interacting with distant tissues and influencing their activity (Durcin et al., 2017; Frühbeis et al., 2015). Despite growing research in this area, the composition of EVs, and particularly how it is modulated by physical activity, remains only partially understood (Ghadami & Dellinger, 2023; Skotland et al., 2020; Haiying Zhang et al., ). Although several phospholipids, such as PC species, were detected in the plasma lipoprotein-free fraction (Dashti et al., 2011), with EVs potentially contributing to their circulating levels, it should be noted that skeletal muscle releases EVs, of which only a small fraction (approximately 1–5%) reach the bloodstream (Guescini et al., 2015; Watanabe et al., 2022). Further investigation into the relative contribution of lipoproteins and EVs, as well as their cellular origin, is needed to clarify the mechanisms driving the lipidome signature influenced by physical fitness in cancer settings.

A limitation of the present study is the small sample size and imbalanced grouping within a population characterised by high intrinsic biological variability due to age, disease status, comorbidities, and medication use (lipid-lowering drugs and antidiabetic medication), whose potential confounding effects were only partially addressed through ANCOVA adjustment. Furthermore, women were not represented in the study, which, although it reflects the higher incidence of GC in men, still represents an important shortcoming. Given the influence of estrogen on lipid metabolism, including women could introduce variability in lipid profiles and potentially confound the interpretation of the results, particularly in small cohorts. The reduced analytical power arising from these constraints limits the robustness and generalisability of the conclusions that can be drawn, reinforcing the exploratory and hypothesis-generating nature of the present findings and the need for validation in larger, prospectively designed and sex-balanced cohorts.

A major strength of our study is the novel identification of associations between circulating lipid species and multiple measures of physical performance status in GC patients, a relationship that, to our knowledge, has not been previously explored. Among the lipid species identified, PI 36:2 and PE plasmalogens demonstrated the strongest associations with physical performance based on 6MWT and other key functional and anthropometric parameters, including limbs circumference, BMI, NRI, handgrip and sit-to-stand test outcomes. Equally noteworthy is the comprehensive and detailed functional and fitness characterization performed in all enrolled patients, which provides a multidimensional assessment of physical capacity that strengthens the clinical relevance and interpretability of the lipidomic findings. Given that physical fitness status can predict GC treatment outcomes (Fukushima et al., 2024; He et al., 2025; Steffens et al., 2019), specific lipidome profiles, including PI 36:2, PE plasmalogens and SM 43:2, may hold value for clinical prognosis. Further studies are needed to validate these findings in larger, prospectively designed, sex-balanced cohorts of patients and healthy individuals with varying levels of physical performance. This approach would allow for a clearer distinction between lipidome signatures driven by physical performance from those induced by cancer or neoadjuvant therapy, facilitating the prediction of treatment complications. Such lipidomic insights could refine preoperative risk stratification and personalize prehabilitation strategies to optimize cancer outcomes.

## Supplementary Information

Below is the link to the electronic supplementary material.

Supplementary file1 (XLSX 54 KB)

Supplementary file2 (TIF 159 KB)—Unsupervised principal component analysis (PCA) (a) and supervised partial least squares discriminant analysis (PLS-DA) (b) score plots

Supplementary file3 (TIF 168 KB)—Statistical analysis of the 11 remaining lipid molecular species from the top 25 lipid species identified on Metaboanalyst (excluding TGs) of the two groups

## Data Availability

Data on lipid species identified across all samples and found to be altered are provided in the Supplementary Information. Additional datasets supporting the findings of this study are available from the corresponding author upon reasonable request.
