# Supplementary figures and images for: Deciphering the circulating lipidome signature associated with physical performance in gastric cancer patients: an exploratory study
Source: Metabolomics. 2026 May 6;22(3):66. doi: 10.1007/s11306-026-02445-1 (PMC13149555; doi:10.1007/s11306-026-02445-1)

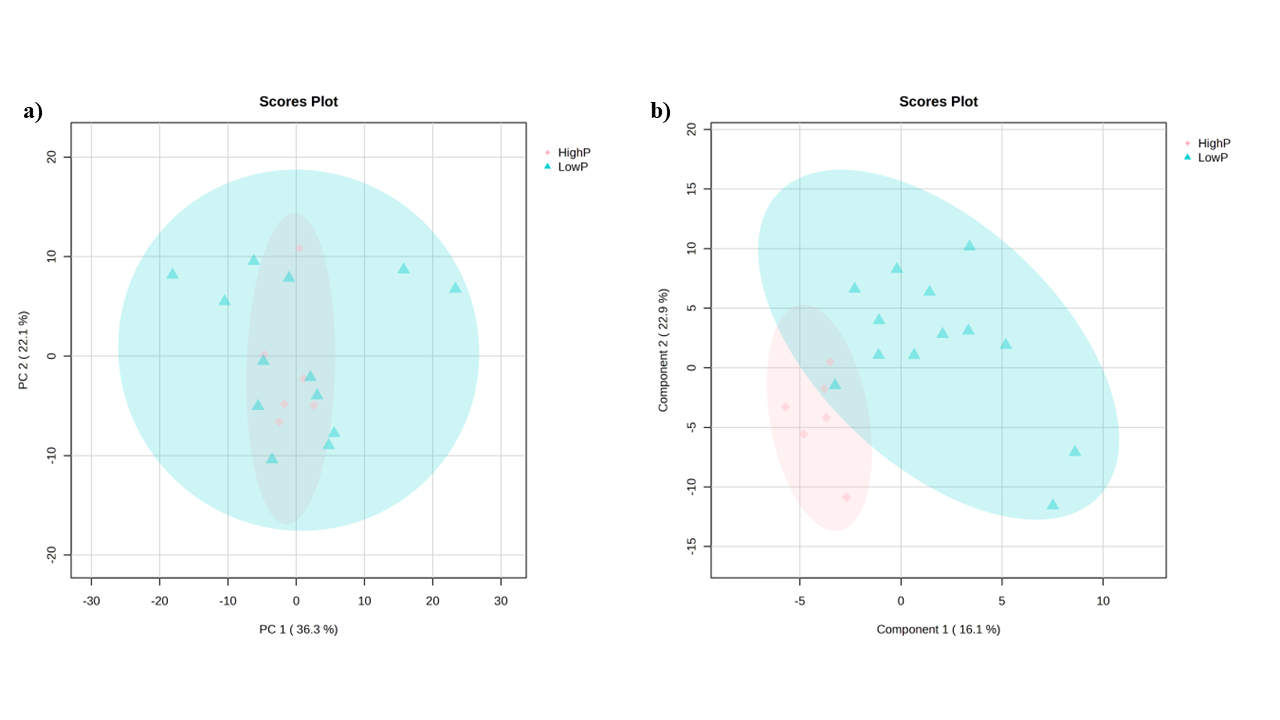

Supplement: Supplementary file 2 — Supplementary file2 (TIF 159 KB)—Unsupervised principal component analysis (PCA) (a) and supervised partial least squares discriminant analysis (PLS-DA) (b) score plots [file 11306_2026_2445_MOESM2_ESM.tif]

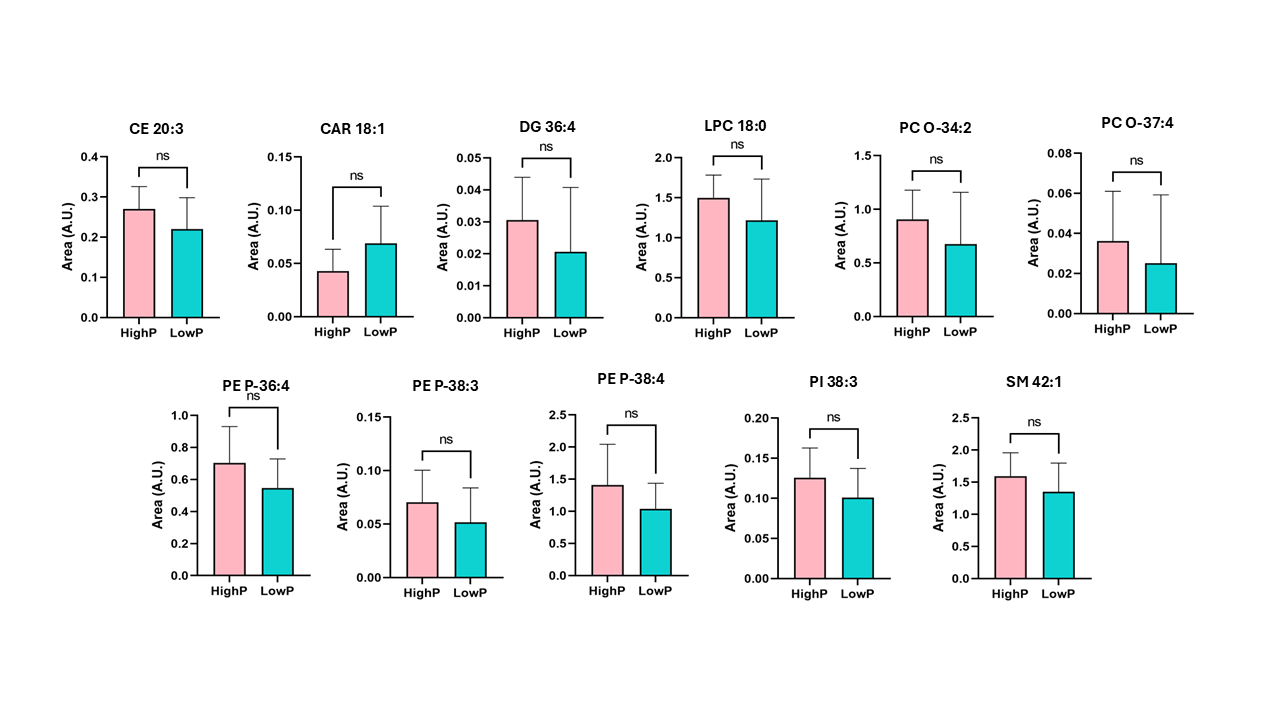

Supplement: Supplementary file 3 — Supplementary file3 (TIF 168 KB)—Statistical analysis of the 11 remaining lipid molecular species from the top 25 lipid species identified on Metaboanalyst (excluding TGs) of the two groups [file 11306_2026_2445_MOESM3_ESM.tif]
